# *‘I Know that I Do Have HIV but Nobody Saw Me’*: Oral HIV Self-Testing in an Informal Settlement in South Africa

**DOI:** 10.1371/journal.pone.0152653

**Published:** 2016-04-04

**Authors:** Guillermo Martínez Pérez, Vivian Cox, Tom Ellman, Ann Moore, Gabriela Patten, Amir Shroufi, Kathryn Stinson, Gilles Van Cutsem, Maryrene Ibeto

**Affiliations:** 1 Médecins Sans Frontières, Cape Town, South Africa; 2 Médecins Sans Frontières Khayelitsha, Cape Town, South Africa; 3 Southern Africa Medical Unit, Médecins Sans Frontières, Cape Town, South Africa; 4 Centre for Infectious Disease Epidemiology & Research, School of Public Health & Family Medicine, University of Cape Town, Cape Town, South Africa; Rega Institute for Medical Research, BELGIUM

## Abstract

Reaching universal HIV-status awareness is crucial to ensure all HIV-infected patients access antiretroviral treatment (ART) and achieve virological suppression. Opportunities for HIV testing could be enhanced by offering self-testing in populations that fear stigma and discrimination when accessing conventional HIV Counselling and Testing (HCT) in health care facilities. This qualitative research aims to examine the feasibility and acceptability of unsupervised oral self-testing for home use in an informal settlement of South Africa. Eleven in-depth interviews, two couple interviews, and two focus group discussions were conducted with seven healthcare workers and thirteen community members. Thematic analysis was done concurrently with data collection. Acceptability to offer home self-testing was demonstrated in this research. Home self-testing might help this population overcome barriers to accepting HCT; this was particularly expressed in the male and youth groups. Nevertheless, pilot interventions must provide evidence of potential harm related to home self-testing, intensify efforts to offer quality counselling, and ensure linkage to HIV/ART-care following a positive self-test result.

## Introduction

Low coverage of HIV-status awareness increases HIV-related morbidity and mortality and prevents infected individuals from using barrier methods to decrease the risk of infecting their sexual partners and from enrolment in HIV-care and prevention of mother-to-child transmission services [1]. In South Africa, according to the 2012 National HIV Prevalence Survey, men were less likely than women to have ever tested for HIV (59,0% vs. 71,5%, p<0,001) [2]. Youth aged 15–24 were also less likely than adults aged 25–49 to have ever received an HIV test (50,6% vs. 78,2%, p<0,001)[2]. Furthermore, in 2012, less HIV-positive men were aware of their status than HIV-positive women (37,8% vs. 55.0%, p<0,001) [2]

To achieve universal ART initiation coverage, testing strategies are a public health imperative in South Africa [3,4]. In eliminating missed opportunities to diagnose HIV-infected individuals, screening programs must be safe and confidential for the population [5]. To meet testing targets, the South African Government began rolling out provider-initiated counselling and testing (PICT) services alongside client-initiated voluntary counselling and testing (VCT) models in 2010 [3,6,7,8]. Opt-out HCT services at antenatal clinics have contributed to reach universal testing coverage among pregnant women, yet innovative strategies are necessary to improve infection disclosure and testing uptake among women’s partners [9,10,11].

HIV-status awareness may be broader if self-testing kits were offered [12]. Home use of HIV self-testing could encourage testing uptake among those individuals who decline PICT or VCT, increase frequency of re-testing among key populations at risk of HIV acquisition, and could facilitate mutual testing with sexual partners [12,13,14].

Home self-testing involves the performance of a HIV test in any private and convenient space. While self-testing is not prohibited by South African regulations, the Department of Health and the South African Medical Association have warned against its use [7,8,15]. A recent qualitative study interviewed South African key stakeholders with influence in national HIV programming reported that self-testing was perceived as having the potential to reach those who do not access HCT; however a lack of counselling, the challenge of ensuring linkage to care, potential abuse of self-testing, and concerns about the accuracy of the devices were noted as barriers to the introduction of HIV self-testing [16]. As of May 2015, in its National HIV Counselling and Testing Guidelines, the Department of Health states that *‘HIV self-testing is currently nor recommended and supported in South Africa’* [4].

Oral HIV self-testing (O-HIVST) is less invasive as it does not require a blood draw [13]. An O-HIVST device distributed in South Africa is the OraQuick ADVANCE HIV-1/2 Rapid Antibody Test (™Orasure Technologies Inc) [17]. In a two-year cluster randomized trial in Malawi, OraQuick showed sensitivity of 93.6% (95%CI:88.2%–97.0%) and specificity of 99.9% (95%CI:99.6%–100%) [18]. In 2015, in KwaZulu Natal, South Africa, OraQuick showed sensitivity of 99.1% (95%CI:96.8–99.9) and specificity of 99.9% (95%CI:99.3–100) [19].

Easy access to home O-HIVST in South African clinics might increase testing uptake among individuals who fear breaches in confidentiality when accessing VCT/PICT [5,7,8,12]. More evidence on acceptability, feasibility, and accuracy of home self-testing in South Africa is necessary [5,7,12]. Concerns around any modality of home self-testing include false-negative or incorrect interpretation of results; self-harm following a HIV-positive test; potentially increased risk of coercive testing to partners and children; and missed opportunities for confirmatory HIV testing and linkage to HIV/ART-care [5,7,13,15,16,19,20,21].

There is paucity of data on coercive testing and linkage with self-testing in South Africa [5,8,19,22]. However, there is research that reports high acceptability and good performance of self-testing; home self-testing was the testing model preferred over PICT and VCT by 22.3% of 466 South Africans in a cross-sectional study [22]. A pilot intervention among healthcare workers in Cape Town using home O-HIVST reported potential for adequate linkage [19]. A usability study reported higher acceptability of O-HIVST compared with fingerstick, but also higher error rates than in previous unsupervised O-HIVST research [23]. In 2015, a feasibility study in KwaZulu Natal reported high agreement in counsellor and participant reading of O-HIVST results and high participant compliance with self-testing procedures [24].

With the purpose of improving knowledge on the potential of self-testing to expand coverage of HIV-status awareness, the primary objectives of this study were to explore reasons for declining PICT or VCT among community members of an informal settlement, and to ascertain the healthcare workers’ and community members’ perspectives on the acceptability of home O-HIVST as an alternative to clinic-based modes of testing.

## Materials and Methods

### Study site

Khayelitsha is an informal settlement located 30 kilometres outside of Cape Town. According to the 2011 Census, Khayelitsha has a population of 391,748 [25,26]. The population is predominantly Black African (99%), the unemployment rate is 38%; and the average monthly income is ZAR 3,200 (approximately 244 USD) [25]. In Khayelitsha, the population can access clinic-based HCT free of charge.

The Wellness Hub is an initiative by Médecins Sans Frontières and the Western Cape Government. The Hub, staffed by counsellors and nurses, is located in the community and offers services for HIV, TB, and STI screening, pregnancy testing, hypertension and diabetes screening, and family planning. All clients that arrive at the Wellness Hub are routinely offered HCT. As of May 2014, only 30% of all patient visits to the Wellness Hub included an HIV test. Our research was conducted at the Site C Wellness Hub of Khayelitsha, which opened in July 2013, and is the second of its kind.

### Study design and period

This study design is mixed-methods research. The first phase is qualitative using thematic analysis [27,28]. Participant sampling started in December 2014, and analysis was finalized in March 2015. The qualitative phase informed a pilot intervention that will offer O-HIVST to Wellness Hub clients who decline HCT. The aim of the pilot phase is to increase testing uptake as well as analyse linkage for clients that use home O-HIVST offered by the Hub counsellor. In this article we report on the findings of the qualitative phase.

### Sampling and recruitment

Research was conducted with three key-informant groups; a) Wellness Hub clients that declined HCT in the past 3 months, c) clients that received HCT and were willing to be interviewed with their partner, and c) nurses, counsellors and community caregivers that provide HIV-care. All participants were aged 18 and above, fluent in either English or Xhosa, and residents of Khayelitsha. Convenience sampling was used to identify healthcare workers. Using Wellness Hub visit registers, a research nurse purposively sampled clients and couples. The nurse contacted telephonically the clients that–according to the registers–met the inclusion criteria and had attended the Wellness Hub in the past three months. The study purpose and the data collection methods were detailed over the phone and individual interviews and group discussions at the Hub were scheduled with all persons that expressed interest. In respect of their autonomous decision, socio-demographic data was not collected from clients that declined to participate in this study.

### Data collection

A semi-structured topic guide was used across couple interviews (CIs), in-depth interviews (IDIs), and focus group discussions (FGDs) (Table 1). Main themes covered were barriers to accept HCT, and issues that related to community acceptability, potential usability, and safety concerns around home O-HIVST. To facilitate examination of their perceptions of the O-HIVST, all participants received a demonstration of the OraQuick O-HIVST [17]. Some participants were invited to participate in both an IDI and a FGD, as we aimed to add an understanding of how social desirability bias could influence their narratives. The topic guide merited adaptation when emerging themes were raised by the participants.

**Table 1:** Interview Topic Guide.

	Main themes explored across data collection
**1.**	Reasons why attendees at the Wellness Hub decline conventional HCT.
**2.**	Potential of O-HIVST to mitigate reasons for community members to decline conventional HCT.
**3.**	Reasons why some individuals in Khayelitsha may not access conventional HCT.
**4.**	Potential of O-HIVST to mitigate reasons for community members not to access HCT.
**5.**	Following OraQuick demonstration: General opinion on O-HIVST.
**6.**	Preference for use of O-HIVST at clinic versus home.
**7.**	Preference for use of counsellor assisted O-HIVST versus unsupervised O-HIVST.
**8.**	Potential benefits of home O-HIVST in Khayelitsha by men and adolescents.
**9.**	Possible scenarios of O-HIVST use among partners.
**10.**	How someone who self-tests positive at home might react?
**11.**	How someone who self-tests negative at home might react?
**12.**	If potential harm is described by the interviewee: measures to reduce adverse events.
**13.**	Best means of post-test support to reach home O-HIVST home users.

In total, eleven IDIs, two CIs, and two FGDs took place at the Wellness Hub, were conducted in English, and lasted 55 minutes on average. All data collection was conducted by a social scientist with experience in Southern Africa (1^st^ author). All participants were provided with a groceries voucher (approximately 9,00USD).

### Analysis and reporting

Data collection and analysis happened iteratively. All recordings were transcribed verbatim by (1^st^ author initials) and any personal identifiers were filtered out. All transcriptions were cross-checked against the recordings. Thematic analysis was done in sequential steps: familiarization with the data by reading the transcripts several times; coding *in vivo* the transcriptions based on a pre-defined coding set; searching, defining and reviewing emerging themes; and then grouping codes into core themes [27,28]. A memo journal and a thematic map were prepared to assist with the analysis. Coding and analysis was done both manually and using software Dedoose. Intercoder reliability was not used.

To improve trustworthiness, the interview topic guide was updated based on initial emerging concepts, and the preliminary findings were triangulated with the FGD participants and with findings from previous research on self-testing. Additionally, deviant cases were analysed, participant words were used in the emerging theory, and our detailed audit trail was examined by peer observers [29].

### Ethics

Written informed consent was sought for each participant to agree for the interview to be tape-recorded. A signed copy of the informed consent documents was given to each participant. All participants were asked to treat discussions as confidential, but the risk that other FGD members might disclose their views to other community members was explained. Once the analysis was finalized, all recordings were deleted to further safeguard anonymity of participants.

This study received ethical approval from the Human Research Ethical Committee of the University of Cape Town (Cape Town, South Africa) and from the Médecins Sans Frontières Ethical Review Board (Geneva, Switzerland).

## Results

This research was conducted in cooperation with twenty participants (Table 2). Six Wellness Hub female clients, who were residents in Khayelitsha and had refused HCT declined participation in this study. These six women were in the age group 21–36 years old. Sampling of participants was discontinued when data saturation was reached.

**Table 2:** Socio-demographic characteristics of research participants.

Pseudonym^#^	Age	Sex	Ethno-linguistic	Occupation	Education	CI*	IDI*	FGD*
**GROUP A:**	**Clients of the Wellness Hub that had declined HCT**							
Laura	41	F	Xhosa	Community worker	Grade 5	-	1	-
Mary	36	F	Xhosa	Domestic worker	Grade 11	-	2	-
Alex	47	M	Xhosa	Watchman	Grade 11	-	3	-
Tom	20	M	Xhosa	Student	Grade 12	-	4	-
Leon	30	M	Xhosa	Unemployed	Grade 11	-	5	-
Arnold	30	M	Xhosa	Watchman	Grade 12	-	6	-
Joseph	40	M	Xhosa	Community worker	Grade 12	-	-	1
Jon	26	M	Xhosa	Journalist	Grade 11	-	-	"
Ryan	30	M	Xhosa	Unemployed	Grade 12	-	-	"
**GROUP B:**	**Health staff supporting the Wellness Hubs**							
Sonia	50	F	Xhosa	Nurse	University	-	-	2
Sylvia	26	F	Xhosa	Counsellor	College	-	7	"
Lea	50	F	Xhosa	Community caregiver	Grade 11	-	8	"
Monica	43	F	Zulu	Nurse	College	-	9	"
Julia	34	F	Xhosa	Counsellor	Grade 11	-	10	"
Carol	37	F	Xhosa	Nurse	College	-		"
Emma	29	F	Xhosa	Community caregiver	Grade 12	-	11	-
**GROUP C:**	**Wellness Hub clients that received HCT and were interviewed with their partners**							
Esther	20	F	Xhosa	Unemployed	Grade 10	1	-	-
Eric	26	M	Xhosa	Courier	Grade 11	"	-	-
Angela	31	F	Xhosa	Driver	University	2	-	-
Ernest	37	M	Xhosa	Planning official	Grade 12	"	-	-

^**#**^ All transcript excerpts in this article are anonymized

* Two Couple Interviews (CIs), 11 In-Depth Interviews (IDIs) and two Focus Group Discussions (FGDs) were conducted

In total, two CIs were conducted with two couples, and eleven IDIs were conducted with six clients and five healthcare workers. Additionally, two FGDs were conducted with four male clients and six healthcare workers. All the healthcare workers were women. Among the couples and the clients, four were women and nine were men. All participants but one had Xhosa as their ethno-linguistic affiliation. All participants lived in Khayelitsha. As the narratives across the CIs, IDIs, and FGDs were quite homogeneous, the word ‘participants’ refers to all three key-informant groups.

### Fear as major reason to decline HCT

Overall, *‘fear’* of knowing one’s HIV-status was highlighted as a major deterrent to accessing HCT. Many individuals were described as not *‘ready’* as they might suspect that they are HIV-infected. This worry was described as more latent among women:

***R*:** *It is men who are overpowering women*, *so it ends up being the men who have five girlfriends and a girl with one boyfriend*. *I think 90% of the women are faithful to their partners*, *yet there are some who are drinking and… but mostly it is men… from my experience*!

*(Monica*, *Wellness Hub nurse interviewed individually*, *43 years)*

To some participants, many individuals were not *‘ready’* because it was common belief people would die of AIDS if they acquired HIV. Some participants explained that most people feared that if they were HIV-infected their partners may *‘neglect’* them, and that they could be *‘stigmatized’* by other community members:

***Ernest*:** *There are people who are still treating people who are diagnosed* [with AIDS] *as outcasts from the community*. *That’s why you find out that some people are still afraid to know their status*, *and some of them are not aware that if you are HIV-positive you still are as healthy as everyone if you are taking the ART… once there is somebody who is diagnosed with HIV*, *the only think that clicks in somebody’s mind is that ‘I am going to die’*.

*(Ernest*, *interviewed with partner*, *37 years)*

Concern to be identified as HIV-infected was described by both clients and healthcare workers. All agreed that the community was excessively preoccupied with others’ problems. HCT was declined by those who anticipated the discomfort of being stared at when leaving the counsellor room.

### Public image in a Xhosa community

The most common sign of discrimination was to hear people gossiping about another person’s HIV status. To a Xhosa, the sense of feeling *‘dignified by society’* was crucial. Thus, community gossip was said to be unbearable. A male client that had been on treatment for TB explained how discrimination could be enacted in public spaces such as shebeens, public buildings that sell alcohol in Khayelitsha:

***Leon*:** *Most of people they go to drink while they take treatment in the shebeens… If they know you are positive*, *some of them laugh*. *But not in front of you*, *like; ‘Hey*! *this guy is HIV’*, *or; ‘His sister is HIV’*. *Not telling him or her face to face… I was one of them*. *I got discriminated*. *Some people laughed at me; ‘Hey*! *Look*, *you are very thin*!*’*.

*(Leon*, *male client interviewed individually*, *30 years)*

The shebeens are spaces where the community capitalize on building their public image. Alcohol abuse fuelled situations of discrimination, but also facilitated the decision to test for HIV in some men. In the FGD with male clients, two men actually requested to take an OraQuick with them, stating that otherwise they would need *‘at least 2 Savannahs* (an alcoholic drink)*’* to test.

The reason men declined HCT included the perception that the clinics exist primarily for women; men are self-conscious of their engagement in risky sexual behaviours; and the social imperative for men to stick to a hegemonic masculinity model. To preserve the public image of a man who is a *‘provider’* for his family, able to bear life’s adversities, and who only goes to the clinic when sick was said to be crucial. This role model was reinforced in the Xhosa youth when they attended the initiation rituals:

***Interviewer*:** *For a Xhosa man*, *what is the ideal public image*?

***Alex*:** *From the culture*, *it means to be strong*, *to have a family*. *To have your things*. *Cattles*. *To get a house*. *The problem about this*, *it’s never been discussed health issues about men*. *The only health issue they know is going to the bush* [circumcision ceremony] *and they come out as man… That they are HIV*, *it’s still a taboo*, *they hear it in the radio*, *they see in the TV…*

*(Alex*, *male client interviewed individually*, *47 years)*

Younger male clients described how their attitudes towards ill-health were shaped by the socially condoned image of what a *‘real’* Xhosa man is. The younger male clients described that their exposure to the social environment of Khayelitsha was affecting how they applied the norms they had been instructed about during the initiation rites. One of the values they tended to forgo was endurance of life’s adversities.

### Lack of trust: another reason to decline HIV testing

Some clients explained that the community could accept home O-HIVST over HCT since a lack of trust in healthcare workers prevented many individuals from accessing HCT. Breaches of confidentiality was mentioned as a reason to distrust healthcare workers. Some clients reported that *‘forced testing’* occurred at the clinics. Arnold, for instance, reported requesting assistance for *‘drop’* (an STI) and was informed that he could not be helped for his presenting complaint if he did not accept HCT. The clients’ bad perception of the counsellors was extended to the quality of counselling services. Alec, a 47-year old male client felt discouraged that the quality of counselling had worsened and explained that *‘It’s not the same as they did before*, *1990s*, *when HIV was stigmatized’*.

### Acceptability of home O-HIVST

Some participants suspected that the most common error people would commit would be to forget to swipe both the upper and lower gums. A few clients suggested that the OraQuick package’s instructions should be provided in Xhosa. Overall, participants agreed that the procedure of OraQuick was easy and that home O-HIVST should be acceptable as many people could benefit from the comfort, confidentiality and security of self-testing in privacy:

***Tom*:** *It’s better because you can take this and go home*. *Nobody sees your results*. *Only you know*. *I prefer that it’s confidential*. *I know that I do have HIV but nobody saw me*, *it’s me alone*. *Now it’s my duty to go there and take the ARVs*.

*(Tom*, *male client interviewed individually*, *20 years)*

A majority of participants distinguished between the acceptability of the OraQuick device and the potential usability of home self-testing. Overall, most participants agreed that acceptability of home O-HIVST would not necessarily mean higher uptake. Some participants believed that O-HIVST could help some individuals overcome their lack of trust in the health establishment because they could self-test at home without the involvement of a healthcare worker.

If home self-testing was offered to the individuals that declined HCT, all participants agreed that it was essential to accompany the distribution of the device with tailored pre- and post-O-HIVST counselling. A few clients and healthcare workers suggested that it would be better to offer O-HIVST in a supervised environment, by first instructing people on the procedure, and then facilitating a place for them to test in privacy. Regardless of the setting where OraQuick was to be offered, many believed that if the individual was not *‘convinced’* of the importance of knowing one’s status, home O-HIVST would not be used by those declining HCT.

Men were mentioned as the Wellness Hub clients that would show most interest in using home O-HIVST. The majority of participants explained how home O-HIVST could assist men to escape from the community gaze in public testing venues. In this manner, men could protect their position in the masculinity role that dictates the requesting of prevention services at the clinic as being health-seeking behaviour associated with women. Albeit perceived as acceptable, easy, comfortable and pragmatic, to many participants, offering an O-HIVST device for home use would not influence certain men to take the decision to self-test if in their inner selves they do not want to know their status:

***Eric*:** *They* [men] *don’t even wanna go to the private clinic*, *mobile clinic… If you give this test*, *they take it and do it to themselves*, *it is gonna be much easy for them*. *Men are very strong physically*, *mentally they are weak*. *Women are strong mentally*, *physically they are weak (laughs)*.

*(Eric*, *interviewed with partner*, *26 years)*

According to a few participants, the youth in Khayelitsha also feared having their public image altered and preferred to spend their time at the shebeens rather than at the clinics seeking prevention services. Based on this perception, the clients and healthcare workers claimed that many youth could be interested in home O-HIVST because some engaged in alcohol abuse and in unprotected sex, tended to distrust their sexual partners, and, though they *‘regretted’* their risky behaviours, many youth did not accept HCT.

### Partners’ use of O-HIVST

There was no consensus with regards to whether the OraQuick should be offered to Wellness Hub clients who claimed they were unaware of their partner’s HIV-status. It was explained that if clients were truly willing to suggest self-testing to their partners, they would rather arrive at home, share their own experience with O-HIVST, and encourage their partners to go to the Wellness Hub. However, it was emphasized that most clients would not even admit to having accepted O-HIVST. As a male client explained, testing with a partner was a *‘risky situation’* and required *‘braveness’*.

Some men manifested interest in taking an OraQuick home with them. The men said that they would not *‘force’* but *‘suggest’* that their partners self-test. The men in the FGD hinted that, if men in Khayelitsha could purchase OraQuick over-the-counter at a pharmacy, some would get it to test their partners. The same participants also insisted it would be better for partners to attend the Wellness Hub to receive pre-O-HIVST counselling before they used an OraQuick.

The participants did not believe coercive testing would be more likely with self-testing than with HCT. Due to lack of trust as a consequence of infidelity, the healthcare workers explained how some women were taking their partners by force to the Wellness Hub and would not stop ‘screaming’ unless they received HCT. Some participants claimed that a few women in Khayelitsha could also exert power over men and insist that they used O-HIVST.

Overall, male dominance over women was noted as a socially condoned gender norm. Manifestations of this norm in daily life were not uncommon in Khayelitsha. The following excerpt from a community caregiver exemplified how some community members imagined that men’s exertion of power over women could be transferred from the domestic arena to the use of home O-HIVST:

***Interviewer*:** *what type of abuse is the most common*?

***Lea*:** *Sexual abuse on the women… beats them when they don’t want to do certain things*.

***Interviewer*:** *About sexual abuse inside the marriage*, *do women perceive that is abuse*?

***Lea*:** *They say that is normal in the marriage*!*… and we tell them; ‘you must be confident with yourself*, *don’t give yourself to a man when you don’t want to’*. *And they say; ‘your man will go outside and look for another woman so it is better for you to give them’*.

***Interviewer*:** *You think they will use the same type of power if they are given this self-test*?

***Lea*:** *Yes*! *I think they will to do the same*, *and force the woman to test*.

*(Lea*, *community caregiver interviewed individually*, *50 years)*

In the scenario where couples used O-HIVST voluntarily, a community caregiver explained that problems could arise if the results were discordant. The two couples interviewed explained how they and other couples could feel if the results were discordant:

***Angela*:** *If he is negative and I am positive and we are at home together*. *I will be very angry and shocked and disappointed*.

***Ernest*:** *…if the people can first be counselled… It is not going to be difficult and it is not going to result in a situation whereby they will fight after they see the results*. *But if they do not have an understanding of counselling*, *then they won’t accept the results*. *And once they don’t accept the results they will start fighting*, *one will start to point the finger on another*, *and I think in those situations some people kill each other…*

*(Ernest and Angela*, *interviewed as a couple*, *37 and 31 years)*

### Education and support to minimize all potential risks

A majority of participants hinted at the potential for confusion if clients were not educated that OraQuick detected antibodies and not the virus in the oral cavity. A few men said that the community believed that exchange of oral fluids did not risk HIV acquisition:

***Alex*:** *Kissing is the best*. *So I think before they know exactly about this*, *they are going to need a lot of training… so that they will trust this*. *That; ‘ok*, *what is it*, *it detects from the mouth*, *you see*?*’*. *Because most believe that the mouth is not that dirty when it comes to HIV*. *We know there are some germs in the mouth but we don't expect the HIV can be detected from the mouth*.

*(Alex*, *male client interviewed individually*, *47 years)*

Likely reactions to a HIV-negative result were examined. Many participants claimed that those who self-tested HIV-negative would not request confirmatory testing. Sexual disinhibition was identified by many as a possible consequence of self-testing HIV-negative. In such scenario, as Jon, a male client, put it: *‘you want to celebrate*!… *you feel released and throw the condom away*!*’*.

The possibility of false negatives should be part of the education given to clients using home O-HIVST. The men in the FGD argued that if the accuracy of finger prick-based self-test were better, this option should be offered rather than the oral-based one. As Jon expressed in the FGD; *‘African men want to know first of all’*.

Potential reactions to an HIV-positive result were examined. Suicide, quarrels and threats at gunpoint were spontaneously mentioned by many participants. A majority knew of quarrels occurring among couples once one is diagnosed as HIV-infected. Only one man actually knew a person who had committed suicide. Two male clients knew of a man who threatened his wife at gunpoint after getting a positive result. However, consensus was reached that violent events would not necessarily be more frequent with home O-HIVST than following clinic-based HCT.

Binge drinking was said to be common after HCT. Hence, all participants perceived that alcohol abuse could be the most likely outcome of testing HIV-positive with home self-testing. The use of *‘tik’* (crystal meth) was considered very unlikely to take place after a HIV-positive result.

Most participants raised the concern that clients who self-tested HIV-positive risked not linking to mainstream HIV/ART-care. All participants agreed that a self-testing program needed to make provision for adequate follow-up of clients who used home O-HIVST.

***Lea*:** *(Self-testing) will be better*, *ne*? *Because it will be him only*. *No me*, *no you*, *no nurse*, *no… only him only*, *and then he can test by himself and then send an SMS*. *He doesn’t know you*, *you see*, *and he will be confident to tell you his status*. *But something that will be happening… the follow-ups… He will not want to follow-up because of the stigma*.

*(Lea*, *community caregiver interviewed individually*, *50 years)*

Visiting clients who failed to communicate their results was not perceived as appropriate due to the fear of being labelled as HIV-infected. The healthcare workers at the FGD stressed that most clients *‘hate home visits’*. A toll-free hotline was the preferred method over text messages, for nurses to question clients about their self-testing result.

The healthcare workers were the most concerned about the type of support that can be provided to minimize the risks of self-testing. According to their narratives, nurses and counsellors should offer OraQuick only if the clients understood all procedures correctly, they manifested willingness to request post-self-testing counselling, and they were reassured that no adverse event would result from the use of self-testing at home. In spite of their concerns, all the healthcare workers agreed with the clients and couples on the benefits of home O-HIVST and in its potential to improve testing uptake among clients declining clinic-based HCT.

## Discussion

The provision of home O-HIVST in South Africa’s public health sector could potentially help overcome men and youth barriers to accept HIV testing. Home O-HIVST may be acceptable as a counsellor-introduced service offered to individuals who decline clinic-based HCT. However, community acceptability could be influenced by the capacity of home self-testing providers to provide timely education and counselling to clients that use O-HIVST.

Acceptability of home O-HIVST could translate into higher uptake among those individuals who do not trust healthcare workers, fear breaches in confidentiality, or who hold concerns of having their public image altered if they access HCT at a healthcare facility. However, acceptability would not necessarily translate in higher uptake among those individuals who feel they are not *‘ready’* to know their HIV-status: those that face difficulties overcoming their *‘fears’* of being neglected by their partners, who worry about suffering from stigma or who fear dying of AIDS.

In agreement with previous research, the community of Khayelitsha shares the perception that the benefits of home O-HIVST are that it is easy to use, that it provides an alternative for clients concerned about intimacy and confidentiality, and that it gives clients control of when and where to self-test [22,23,30]. On the other hand, the participants mentioned some of the hypothesised disadvantages of self-testing that have been outlined in the literature. Namely, concerns on the possible coercive use of self-testing, confusion that OraQuick is an antibody assay that detects HIV-infection in the oral cavity, engagement in sexual disinhibition after a negative result, refusal to request a confirmatory test, and barriers to access post-test counselling and linkage to care [5,13,16,19,20,21]. Currently, there is no evidence on the occurrence of these adverse events in a real home O-HIVST scenario in South Africa. Detailed programme monitoring is required to ascertain whether these concerns will become actual harms that are more prevalent with unsupervised O-HIVST than with HCT.

Throughout all interviews issues around violence were raised. No participant implied that violent events would be more prevalent following home O-HIVST that following HCT. Participants were probed to further narrate their views on how violence in Khayelitsha was normalized. Exposure to cases of violence had influenced the participants to suggest that any worst case scenario might be feasible, and thus associated HIV testing in the domestic scenario with the potential for violence. The healthcare workers confirmed that these were assumptions influenced by the awareness of participants of the harms that might result from clinic-based HCT. High rates of murder, attempted murder, domestic violence, and sexual assault as reported to the South African Police Service in Khayelitsha (SAPS) influenced community members to feel unsafe, anticipate worst case scenarios, and normalized mentioning violence events in their daily life conversations [27,31,32]

### Implications for practice

This qualitative research has informed a pilot intervention at the Wellness Hub in Khayelitsha. Suggestions made by the participants were integrated into the standard operating procedures that guide counsellors to introduce home O-HIVST for clients that decline HCT at the Hub. Information, education, and communication materials are distributed in Xhosa, and only after each O-HIVST client receives pre-test counselling from the counsellors. All education emphasizes HIV transmission pathways, the concept of antibody-based testing, the window period, and the need to continue using condoms. The counsellors provide detailed guidance on how to link to post-O-HIVST care. The follow-up of users of home O-HIVST involves enquiring about their results via mobile phone technologies and monitoring the incidence of unintended harms. One of the pilot intervention’s main objectives is to measure linkage to care among those clients that self-test HIV-positive.

### Limitations

Limitations of this study are its small sample size and its lack of generalizability. However, the aim of this qualitative research was not to achieve findings that are transferable to the general population [29], but rather to identify generic social processes that could be further explored in qualitative inquiry in similar contexts in South Africa and globally [33].

Maximum variation sampling was pursued; similar representation of women and men, age ranges, and education levels representative of the study population was achieved. Nevertheless, a majority of female participants in this research were healthcare workers, as six Wellness Hub female clients declined to participate. In respect of their autonomy, we did not ask these women to explain their reasons for declining participation. To minimize harms derived from this research, and considering the described fears of being labelled as HIV-infected, we opted not to insist on recruiting females–who had declined HCT at the Hub. Another reason for discontinuation of female client sampling was that data saturation was reached. Furthermore, relying on only four female clients as participants was a partial limitation to explore the topic under study, as the six female healthcare workers were all residents of Khayelitsha, all Xhosa, and most shared the female clients’ and couples’ perspectives on O-HIVST both as HIV-care providers and as self-reported clients of HCT services.

The narratives across the key-informant groups were very homogeneous; hence our analysis was precluded from an occupational point of view. However, it was significant that healthcare workers raised more concerns of potential harms. It needs to be further explored if healthcare workers’ concerns, which are not grounded in evidence of actual adverse events, will become barriers to the provision of home self-testing in informal settlements such as Khayelitsha.

Another limitation was the use of thematic analysis. A higher level of interpretation could have been reached if we had used other inductive approaches such as constructivist grounded theory [34]. However, our aim was to explore how the participants’ viewpoints could have implications for practice (e.g. inform the pilot stage) rather than co-generating theory with their collaboration. Future research using interpretive qualitative approaches will be helpful to add more light on community members’ potential barriers to request post-test counselling and to link to care following a positive home O-HIVST.

### Future prospects

To what extent populations could potentially disengage from counsellor-introduced O-HIVST services due to perceptions of the quality of healthcare is a concern that can be addressed via continuing education programs that emphasize compliance with privacy, autonomy, and confidentiality principles. These principles must be enacted in public healthcare facilities for people to overcome barriers to access home O-HIVST. Advocacy needs to tackle the community’s perception towards healthcare workers by creating awareness on their contribution in facilitating access to HIV/ART-care.

The partial understanding of HIV transmission pathways, the concepts of antibodies, or the window period, are findings that deserve further exploration in the frame of quantitative research. The extent to which key populations could be prevented from accessing O-HIVST services, and TB or STI-care in the clinics as consequence of what they might perceive as *‘forced’* HCT needs further examination. In spite of the overall acceptability of O-HIVST as evidenced by this research, the community must be confident that they would not be requested to accept services others than home O-HIVST against their will.

Our findings suggest that home O-HIVST has the potential to help hard-to-reach populations overcome barriers to test for HIV. Men and youth are identified as clients who could benefit from having access to clinics that provide home O-HIVST services. Despite not being within the scope of our research, a description of what the hegemonic Xhosa masculinity model emerged from our data collection [35]. The gendered norms that are associated to this masculinity model that most Xhosa male adhere to were described as contributors to men failing to accept HCT in primary healthcare clinics that are perceived by men as spaces for women [36]. South African key stakeholders agreed with our participants that self-testing could help address current gender disparities in HIV testing uptake, by affording men the opportunity to act autonomously, to make decisions on their health privately, and at their own convenience [16]. We recommend that future research examine how current masculinity models can be capitalized on improving accessibility, and uptake of unsupervised O-HIVST among adult men and male youth as key populations in HIV testing programmes.

## Conclusion

This research frames barriers and opportunities for accepting home O-HIVST in South Africa. Community members and healthcare workers were probed to explore how the benefits of home O-HIVST could potentially outweigh its perceived potential risks, and their narratives supported that home O-HIVST would not necessarily increase vulnerabilities in a greater manner than conventional HCT does. This research adds evidence of the need for self-testing service providers to ensure the five ‘Cs’ as outlined by the World Health Organization are guaranteed: consent, confidentiality, the opportunity for counselling, correct results, and linkage to care [37]. Furthermore, any pilot home O-HIVST intervention must assess how this innovation contributes to completion of all steps of the cascade of HIV-care, from testing, linkage to care, treatment, long-term retention in care, and a suppressed viral load.

In applying the knowledge this research contributes to programme implementers must consider that efforts are necessary in ensuring that future documented evidence of harms and benefits informs the counselling, support, and monitoring components of home self-testing. Makusha et al. recommend not to restrict self-testing in South Africa based on fears of harms, but rather enhance programmatic monitoring of unintended harms [16]. In attempting to follow-up the impact of home O-HIVST usage it should be remembered that the rationale for offering this testing alternative is to allow choice for users to test without the need for a health worker to be present. Should enforced follow-up at government clinics represent a barrier that could undermine the principal motivation for those seeking self-testing–namely full confidentiality–, service programmers should reconsider the level of monitoring they would like to impose to the users.
